# A High-Resolution VOC Emission Inventory for Gas Stations in a Typical Yangtze River Delta City: Implications for Ozone Formation, Secondary Organic Aerosol Formation, and Health Risks

**DOI:** 10.3390/toxics14060486

**Published:** 2026-06-01

**Authors:** Tianyu Chen, Xinmei Zheng, Chunlei Liu, Ming Wang, Fangjian Xie, Jing Li

**Affiliations:** 1 Collaborative Innovation Center of Atmospheric Environment and Equipment Technology, Jiangsu Key Laboratory of Atmospheric Environment Monitoring and Pollution Control, School of Environmental Science and Engineering, Nanjing University of Information Science & Technology, Nanjing 210044, China; 202283300701@nuist.edu.cn; 2 Nanjing Municipal Academy of Ecological and Environmental Protection Sciences, Nanjing 210046, China; zheng_xinmei@163.com (X.Z.); leilananjing@163.com (C.L.); strong886@126.com (F.X.); 3 Institute of Child and Adolescent Health, School of Public Health, Peking University, Beijing 100871, China; jing.li@hsc.pku.edu.cn

**Keywords:** VOC emission inventory, gas station, health risk, ozone, secondary organic aerosol

## Abstract

Gasoline evaporation is a significant source of urban volatile organic compounds (VOCs). In this study, we selected Nanjing, a major city in the Yangtze River Delta of China, and developed a high-resolution (1 km × 1 km) gridded VOC species emission inventory for gas stations based on measured VOC emission characteristics and statistical data on gasoline and diesel sales. The results show that VOC emissions from gas stations were correlated with population density and road networks, and were mainly concentrated in the downtown area. The emitted VOCs were dominated by alkanes (58%) and oxygenated VOCs (19%), with i-pentane, n-butane, and methyl tert-butyl ether (MTBE) as the major components. C4–C5 alkenes were identified as the key contributors to ozone (O_3_) formation, while aromatics contributed most to secondary organic aerosol (SOA) formation. Health risk assessment indicates that, for gas station workers, both carcinogenic and non-carcinogenic risks associated with gasoline and diesel VOC evaporation exceed acceptable thresholds. Benzene, 1,2-dichloroethane, and 1,2-dibromoethane posed the highest carcinogenic risks, whereas acrolein, benzene, and 1,3-butadiene contributed most to non-carcinogenic risks. For urban residents, the health risks from gas station VOC emissions were generally within acceptable levels; however, under unfavorable meteorological conditions, residents living near gas stations may still face elevated health risks. This study highlights the significant impacts of gas station-related VOC emissions on air quality and human health, and informs targeted control and mitigation strategies for gasoline evaporation.

## 1. Introduction

Ambient volatile organic compounds (VOCs) are key precursors to both ozone (O_3_) and fine particles (PM_2.5_) [1], which have become major barriers to further air quality improvement in China [2,3]. Urban atmospheric VOCs arise mainly from anthropogenic sources, such as industrial processes, solvent use, vehicular exhaust, and fuel evaporation [4]. Previous studies have shown that traffic-related sources, particularly vehicular exhaust and gasoline and diesel evaporation [5,6], are important contributors to urban VOCs in China [7]. Over the past two decades, China has implemented stringent vehicle emission controls [8]. Since July 2023, the China VI Stage 6b emission standard has been fully implemented nationwide [9]. Under this standard, the non-methane hydrocarbon (NMHC) emission limit is 35 mg·km^−1^, corresponding to reductions of 97% relative to the China I standard and 42% relative to Stage 6a [10], thereby substantially lowering VOC emissions from vehicular exhaust [11]. In parallel, China has issued the Technical Requirements for Online Monitoring Systems of Vapor Recovery at Gas Stations [12], which require gas stations to install vapor recovery systems for refueling and tank breathing losses, with recovery efficiencies exceeding 80% [13]. However, the actual control efficiency of these vapor recovery systems ranged from 63% to 85% [14]. Existing studies suggest that evaporative emissions from gas stations still account for approximately 10–20% of urban atmospheric VOCs [15,16,17], and this contribution may be even higher in densely populated urban centers [18]. Therefore, accurately quantifying VOC emissions from gas stations is essential for assessing their impacts on ambient air quality and human health [19,20,21].

VOC emissions from gas stations are characterized by complex chemical compositions [22] and widespread spatial distribution. Previous studies have investigated the chemical profiles of gasoline evaporative VOCs through headspace experiments [23,24] and on-site sampling at gas stations [25,26]. These studies showed that C4–C5 alkanes were the dominant components in gasoline headspace vapors, while alkenes, oxygenated volatile organic compounds (OVOCs), and aromatics also contributed substantially. Although several studies have developed VOC emission inventories for gas stations in cities such as Beijing, China [27], and Mashhad, Iran [28], based on the U.S. Environmental Protection Agency (EPA) Compilation of Air Pollutant Emission Factors (AP-42), most studies focused on total VOC emissions at the city scale [29,30]. Given the widespread distribution of gas stations, their VOC emissions would exhibit considerable spatial heterogeneity. Therefore, it is essential to establish high-spatial-resolution speciated VOC emission inventories and quantitatively assess their contributions to O_3_ and secondary organic aerosol (SOA) formation, as well as their potential impacts on human health.

This study focuses on Nanjing, a major city in the Yangtze River Delta region, to quantify VOC emissions from individual gas stations by integrating site-specific gasoline and diesel sales data with measured VOC chemical compositions. A high spatial resolution gridded (1 km × 1 km) emission inventory of speciated VOCs for gas stations was developed using the Kriging interpolation method. Based on this inventory, the O_3_ formation potential (OFP) and secondary organic aerosol formation potential (SOAP) of gas station VOC emissions were evaluated, and the carcinogenic and non-carcinogenic health risks to gas station workers and nearby residents were assessed. These results improve understanding of the environmental and health impacts of gas station VOC emissions and inform targeted control and mitigation strategies for gasoline evaporation.

## 2. Materials and Methods

### 2.1. VOC Sampling and Chemical Analysis at Gas Stations

VOC sampling was conducted from July to September 2024 at three large gas stations located in densely populated urban areas, each with an annual fuel sales volume of approximately 10,000 tons. Large stations also generally have more stable and continuous fuel turnover, reducing the influence of fuel aging or storage-related variability. The summer sampling period was selected because high ambient temperatures during summer can enhance gasoline and diesel evaporation, resulting in elevated emission factors [31].

Air samples influenced by gasoline or diesel evaporation were collected about 20 cm downwind of the fuel nozzle using pre-cleaned and evacuated 3.2 L fused silica-lined stainless-steel canisters (Entech Instruments, Inc., Simi Valley, CA, USA). This distance was selected to approximate the worker’s personal breathing zone during refueling, which is generally defined as the area within approximately 25 cm of the nose and mouth according to the standard occupational hygiene protocols established by the U.S. National Institute for Occupational Safety and Health [32]. Therefore, the selected distance can better represent near-field inhalation exposure while reducing dilution from ambient air. Sampling was conducted at approximately 1.5 m above ground, within the typical human breathing zone, to better represent the breathing-zone exposure during refueling. Similar to industrial hygiene and occupational exposure assessments for short-term peak exposures [33], instantaneous sampling (lasting approximately 2 min) was adopted because vehicle refueling is a short, episodic process, typically lasting about 3–5 min.

Before field deployment, all canisters were cleaned and evacuated to <20 mTorr using high-purity nitrogen through three repeated cycles using the canister cleaner (Entech 3100, Entech Instruments, Simi Valley, CA, USA). Canister cleanliness was verified by randomly selecting one canister from each batch, filling it with high-purity nitrogen, and confirming that the concentrations of target VOCs were below the method detection limits (MDLs). The vacuum integrity of the canisters was also checked before sampling to prevent leakage. After sample collection, the canisters were transported to the laboratory and analyzed within one week. A total of ten samples were collected, including three from No. 92 gasoline, two from No. 95 gasoline, two from No. 98 gasoline, and three from diesel.

VOCs were pre-concentrated using a low-temperature pre-concentration system (7200, Entech Instruments, Inc., Simi Valley, CA, USA) and subsequently analyzed using gas chromatography coupled with flame ionization detection and mass spectrometry (GC-FID/MS, 7890B-5977A, Agilent, Santa Clara, CA, USA) [34]. Briefly, the Entech 7200 pre-concentrator enriches VOCs from canister air samples using a three-stage cryogenic trapping process. The first two stages remove major matrix components such as water vapor and CO_2_ while concentrating the target VOCs, and the final cryogenic focusing stage further narrows the analyte band before thermal desorption and transfer to the GC–FID/MS system for chromatographic separation and detection of VOCs. Using a Dean’s switch, C2–C3 hydrocarbons were separated using a PLOT (Al_2_O_3_/KCl) column (30 m × 0.25 mm × 3.0 µm, J&W Scientific, Folsom, CA, USA) and detected by FID, whereas the remaining VOC species were separated using a DB-624 column (60 m × 0.25 mm × 1.8 µm, J&W Scientific, Folsom, CA, USA) and detected by MS. Two commercial VOC standard gas mixtures (55-NMHC and TO-15; Spectra Gases Inc., Branchburg, NJ, USA) were used to calibrate target VOCs. A total of 101 species were quantified, including 28 alkanes, 12 alkenes and alkynes, 17 aromatics, 34 halocarbons, and 10 oxygenated VOCs. To ensure data quality, routine quality assurance and quality control (QA/QC) procedures were implemented, including regular instrument maintenance; hourly checks of internal standard responses using bromochloromethane, 1,4-difluorobenzene, and 1-bromo-3-fluorobenzene for internal calibration; and daily analyses of 2 ppb standard gas mixtures for routine calibration. The five-point calibration curve (0.5, 2, 4, 8, and 16 ppbv) showed good linearity for all target species (R^2^ > 0.99). The MDLs of individual VOCs were determined as the concentrations corresponding to a signal-to-noise ratio of 5, and ranged from 0.009 to 0.059 ppbv.

### 2.2. Calculation of VOC Emissions from Gas Stations

#### 2.2.1. Calculation of Total VOC Emissions

In this study, VOC emissions from gasoline at gas stations were calculated using the emission factor (*EF*) method (Equation (1)). Five emission sources were considered: (1) emissions from tanker unloading, (2) emissions from vehicle refueling, (3) emissions from storage tank breathing, (4) emissions from fuel nozzle dripping, and (5) emissions from fuel hose permeation.

(1) $$E=\frac{A}{\rho }\times [\sum_{i=1}^{5}UEFi\times (1-\eta i)]/1000$$

where *E* represents the total VOC emissions from gasoline (kg); *i* denotes a specific VOC emission source; *A* is the gasoline sales volume (kg); *ρ* is the gasoline density, assumed to be 0.75 g·mL^−1^ [35]; *UEF_i_* is the uncontrolled emission factor of source *i* without control measures (mg·L^−1^); and *η_i_* is the VOC emission control efficiency for source *i*.

Gas stations were classified into three types based on annual gasoline sales volume. Type I stations have annual sales below 2000 tons and are equipped with Stage I (S1, vapor recovery during tanker unloading) and Stage II (S2, vapor recovery during vehicle refueling) systems. Type II stations have annual sales of 2000–5000 tons and, in addition to S1 and S2 systems, are equipped with an online VOC monitoring system (OMS). Type III stations have annual sales exceeding 5000 tons and, besides the configurations of Type II stations, are further equipped with a tertiary vapor recovery device (VRD).

Table 1 summarizes the *UEF*, *η*, and *EF* (i.e., *UEF* × (1 − *η*)) values for each source for the three types of gas stations. For gasoline evaporation sources (1), (3), (4), and (5), the *UEF* values were taken from the recommendations by the California Air Resources Board (CARB) [36], while the *η* values were updated according to actual conditions in Nanjing [37]. For source (2) (vehicle refueling), the *UEF* depends on both the vapor recovery system installed at the station and the penetration of onboard refueling vapor recovery (ORVR) systems in vehicles. Given that ORVR has been required for light-duty gasoline vehicles in Nanjing since 2019, the ORVR penetration rate was adjusted from 15% [37] to 25% in this study, resulting in a UEF of 768 mg·L^−1^ for vehicle refueling. As shown in Table 1, Type I stations exhibited the highest VOC *EF* (311 mg·L^−1^), which is 1.7 and 2.2 times those of Type II and Type III stations, respectively. At Type I stations, the vehicle refueling process exhibited the highest *EF* (179 mg·L^−1^), approximately three times that of nozzle dripping and tanker unloading. For Type II stations, the vehicle refueling process also shows the highest EF (90.9 mg·L^−1^), about 1.6 and 3.3 times those of the nozzle dripping and tanker unloading, respectively. At Type III stations, the EF values for vehicle refueling and nozzle dripping were comparable, with both at around 50 mg·L^−1^.

Diesel has a lower vapor pressure than gasoline, and studies on evaporative VOC emissions from diesel remain limited. In this study, the VOC emissions from diesel evaporation were estimated by multiplying diesel sales volume by the *EF* reported in the literature (0.08 kg·t^−1^) [38].

**Figure 1:**
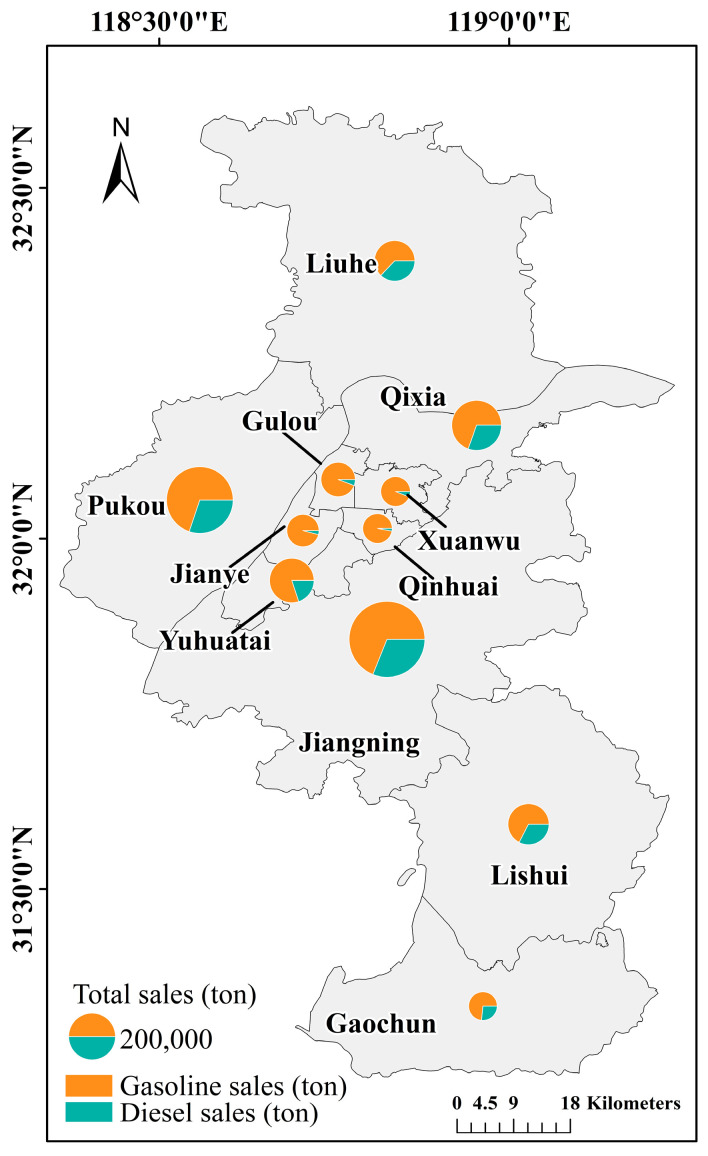
Spatial distribution of gasoline and diesel sale volumes across districts in Nanjing during 2023.

In 2023, 321 gas stations were operating in Nanjing, with total gasoline and diesel sales of 1.61 × 10^6^ tons and 5.40 × 10^5^ tons, respectively. Type I, Type II, and Type III stations accounted for 15%, 28%, and 57% of all stations, respectively. Figure 1 shows gasoline and diesel sales at individual stations. Based on the 2023 sales data, station-level VOC emissions from gasoline and diesel were estimated. These emissions were subsequently allocated to 1 km × 1 km grids according to station coordinates, and the spatial distribution of VOC emissions from gas stations was derived using Kriging interpolation.

#### 2.2.2. Calculation of VOC Emissions for Individual Species

Emissions of individual VOC species were estimated from the total VOC emissions of gas stations (*E*) using the measured chemical composition profiles of gasoline and diesel VOCs obtained in this study:

(2) Ei=E×Pi

where *E_i_* represents the emissions of VOC species *i* (kg), and *P_i_* is the mass fraction of species *i* in the total VOCs, which was calculated by first converting the observed mixing ratio (ppbv) of each species to mass concentration (μg m^−3^), and then dividing the mass concentration of species *i* by the total VOC mass concentration.

### 2.3. Estimating O_3_ and SOA Formation Potential

The potential impacts of VOC emissions from gas stations on O_3_ and SOA were evaluated using O_3_ formation potential (OFP) and SOA formation potential (SOAP):

(3) $$OFP=\sum_{i=1}^{n}Ei\times MIRi$$

where *MIR_i_* is the maximum incremental reactivity of VOC species *i* (kg O_3_·kg^−1^ VOC). The values were obtained from the previous literature [39].

(4) $$SOAP=\sum_{i=1}^{n}Ei\times FACi$$

where *FAC_i_* is the SOA formation coefficient of VOC species *i* (kg SOA·kg^−1^ VOC). The values were obtained from previous literature [40,41].

### 2.4. Health Risk Assessment

The potential health impacts of VOC emissions from gas stations to both station workers and nearby residents were evaluated using the excess lifetime cancer risk (ELCR) and hazard quotient (HQ) for individual species. The total ELCR (TELCR) and hazard index (HI) were calculated as follows:

(5) $$TELCR=\sum_{i=1}^{n}ELCR_{i}=\sum_{i=1}^{n}EC_{i}\times UR_{i}$$

(6) $$HI=\sum_{i=1}^{n}HQ_{i}=\sum_{i=1}^{n}\frac{EC_{i}}{RfC_{i}}$$

where *UR_i_* represents the unit inhalation cancer risk of VOC species *i* (mg^−1^·m^3^), with values obtained from the U.S. EPA Integrated Risk Information System (IRIS) [42] and the California Office of Environmental Health Hazard Assessment (OEHHA) [43]; and *RfC_i_* is the reference concentration for VOC species *i* (mg·m^−3^), with values taken from the IRIS [42] and the Agency for Toxic Substances and Disease Registry (ATSDR) [44]. The specific *UR_i_* and *RfC_i_* values used for the health risk assessment are presented in Table S1 of the Supplementary Materials. *EC_i_* is the exposure concentration of VOC species *i* (mg·m^−3^), which was calculated based on the U.S. EPA exposure model [45]:

(7) $$EC_{i}=\frac{CA_{i}\times ET\times AD\times ED}{AT}$$

where *CA_i_* represents the concentration of VOC species *i* resulting from gasoline or diesel evaporation at the station (mg·m^−3^). *ET*, *AD*, and *ED* denote the daily exposure hours, annual exposure days, and exposure duration years, respectively, while *AT* is the averaging time (hours), corresponding to the expected lifetime.

For gas station workers, *CA_i_* was taken as the mean measured concentrations at the station. *ET* values were estimated based on typical working hours, fuel consumption structure, and refueling frequency. The ET for gasoline was set to 1–8 h, with the upper limit corresponding to the standard 8 h working shift used in occupational exposure assessment in China [46]. For diesel, a lower ET range of 0.5–4 h was adopted because diesel refueling occurs less frequently at urban gas stations, as diesel is mainly used by heavy-duty vehicles [47] and is affected by daytime traffic restrictions. *AD* and *ED* were set at 250 days and 35 years, respectively, for both fuel types [48]. For Nanjing residents, considering continuous VOC emissions from gas stations, *EC_i_* was assumed to be equal to *CA_i_*, which was estimated using a box model assuming Nanjing to be a well-mixed box [49]:

(8) $$CA_{i}=\frac{L\times \tau_{i}}{(u\tau_{i}+L)\times H}\times \frac{E_{i}}{S}$$

where *L* is the box length (m), determined by the geographic setting of Nanjing and the prevailing annual wind direction (southeast–northwest) [50], and set to 1 × 10^5^ m; *u* is the average wind speed in Nanjing, taken as 2.20 m·s^−1^, obtained from the Environmental Meteorological Data Service Platform (http://eia-data.com (accessed on 1 March 2025)); *H* is the mixing-layer height, set to 1490 m in summer and 290 m in winter based on observation studies [51]; and *E_i_* is the emission rate of VOC species *i* from gasoline or diesel evaporation (mg·s^−1^). Assuming no significant seasonal variation in fuel consumption, the *E_i_* values for summer and winter in 2023 were estimated according to the seasonal variation in VOC emission factors for gas stations [31]. *S* is the area of the study region (m^2^), set to 6.59 × 10^9^ m^2^ for Nanjing and 3.95 × 10^8^ m^2^ for the central urban area. Assuming that reaction with hydroxyl radical (OH) is the dominant removal pathway for VOCs, the atmospheric lifetime (*τ*, s) can be calculated as follows:

(9) $$\tau =\frac{1}{k_{OH}\times [OH]}$$

where *k_OH_* is the reaction rate constant of VOCs with OH (cm^3^·molecule^−1^·s^−1^), with values obtained from the Master Chemical Mechanism (MCM); and [*OH*] denotes the average OH concentration, which was assumed to be 5 × 10^6^ molecule·cm^−3^ in summer and 1 × 10^6^ molecule·cm^−3^ in winter according to the published literature [52].

## 3. Results

### 3.1. Chemical Composition of VOC Emissions from Gasoline and Diesel Evaporation

**Figure 2:**
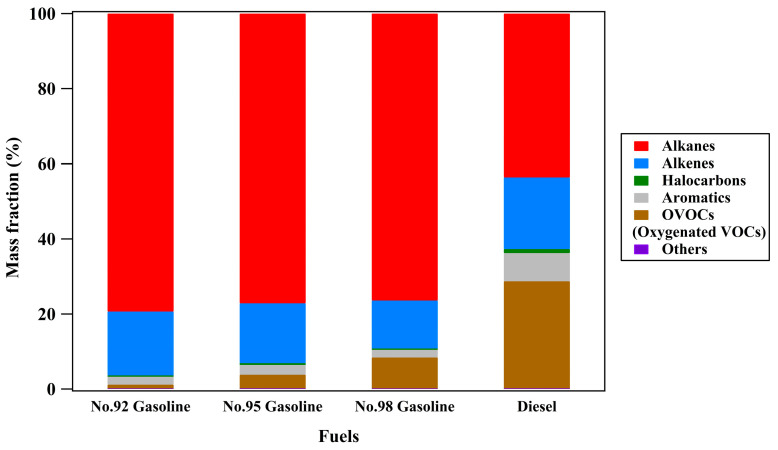
Mass fractions of different VOC categories to total measured VOC concentrations for No. 92, No. 95, and No. 98 gasoline, and diesel.

Figure 2 shows the relative contributions of different VOC classes to the measured mass concentration of VOC emissions from gasoline and diesel at gas stations. Alkanes dominate gasoline evaporative VOCs, accounting for more than 75% of total VOC mass in No. 92, No. 95, and No. 98 gasoline, followed by alkenes (13–17%), aromatics (2–3%), OVOCs (1–8%), and halocarbons (0.34–0.46%). These results are generally consistent with previous studies on refueling emissions [53], with a composition ratio of approximately 70:14:1:13 for alkanes, alkenes, aromatics, and OVOCs. However, some differences were observed compared with gasoline vapors measured in headspace experiments, with 59–72% reported for alkanes, 18–28% for alkenes, and 4–10% for aromatics [24]. Compared with the refueling emissions reported in Japan in 2015 [54], which consisted mainly of alkanes (80%) and alkenes (20%), the results of this study indicate higher contributions of aromatics and OVOCs in China. The low contributions of halocarbons observed in this study have also been reported in other gas station measurements in China. For example, Zeng et al. (2022) reported halocarbons in evaporative VOC source profiles from gas stations in Guangzhou, with a higher contribution of 5.5 ± 1.2% [55]. Sun et al. (2024) also detected halogenated VOCs in source profiles from gas stations in Zhengzhou [56].

The chemical composition of VOCs from diesel evaporation differed significantly from that of gasoline. In diesel VOC emissions, alkanes accounted for 44%, lower than in gasoline, whereas the contributions of OVOCs and aromatics were substantially higher, reaching 29% and 8%, respectively. The alkene contribution (19%) was also slightly higher than that in gasoline. The diesel VOC composition observed in this study differed from previous reports on diesel evaporative VOCs [57], in which aromatics and alkanes dominated diesel evaporative VOCs, together accounting for 89%, with aromatics alone contributing 42%, while alkenes accounted for only 1%. These differences may be the result of variations in diesel refining processes.

**Figure 3:**
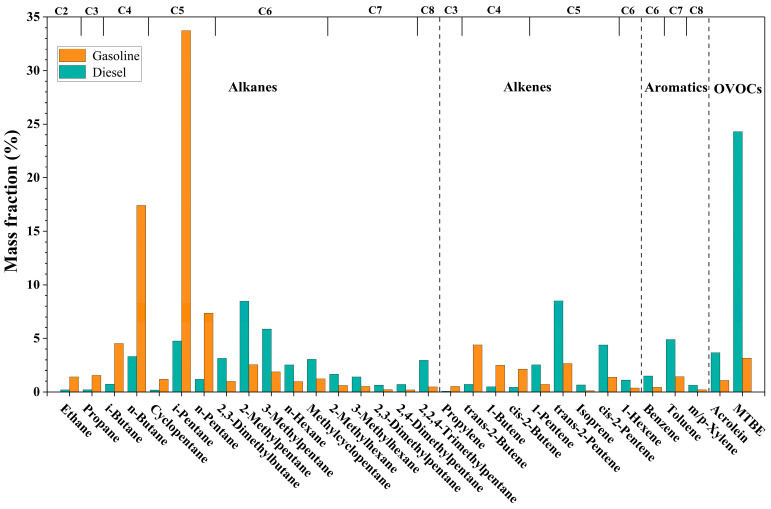
Chemical profiles of VOCs from gasoline and diesel evaporation. Only the 31 major species with mass fractions above 0.5% were shown, including 17 alkanes, 9 alkenes, 3 aromatics, and 2 oxygenated VOCs (OVOCs).

Figure 3 shows the contributions of individual VOC species to the total measured VOC concentration from gasoline and diesel emissions. Gasoline VOC emissions were dominated by C4–C6 alkanes, with i-pentane as the largest contributor (27%), followed by n-butane (14%). In diesel VOC emissions, methyl tert-butyl ether (MTBE) was the largest contributor (24%), followed by trans-2-pentene (9%). These findings are consistent with those reported for gas stations in Guangzhou [55], where MTBE was also identified as a key component in both gasoline and diesel evaporative VOCs. The higher alkene contribution in diesel VOCs than in gasoline may be related to the strict alkene content limits in the China VI gasoline standard. Comparing the gasoline and diesel evaporative source profiles obtained in this study with those reported in U.S. and Europe revealed several differences. In the headspace vapor of U.S. reformulated gasoline, VOCs were mainly dominated by ethanol and alkanes, while MTBE was rarely detected because it had been phased out due to its high potential for groundwater contamination [58]. However, MTBE is still widely used in Chinese gasoline as an oxygenated additive to improve octane number and combustion efficiency [59]. In contrast, consistent with our results, recent observations at an Italian gasoline station also found that MTBE and toluene remained important contributors to VOCs from gasoline evaporation, whereas the benzene contribution was lower than that in our study, possibly due to the stricter benzene limits imposed by the European Union [60]. In this study, the mass fractions of acrolein for gasoline and diesel evaporation were 1.07% and 3.67%, respectively. Another study in China also reported trace acrolein in ambient air surrounding gas stations [61]. However, acrolein was not detected in the U.S. gasoline headspace study [58].

### 3.2. VOC Emission from Gas Stations and Its Spatial Distribution

**Figure 4:**
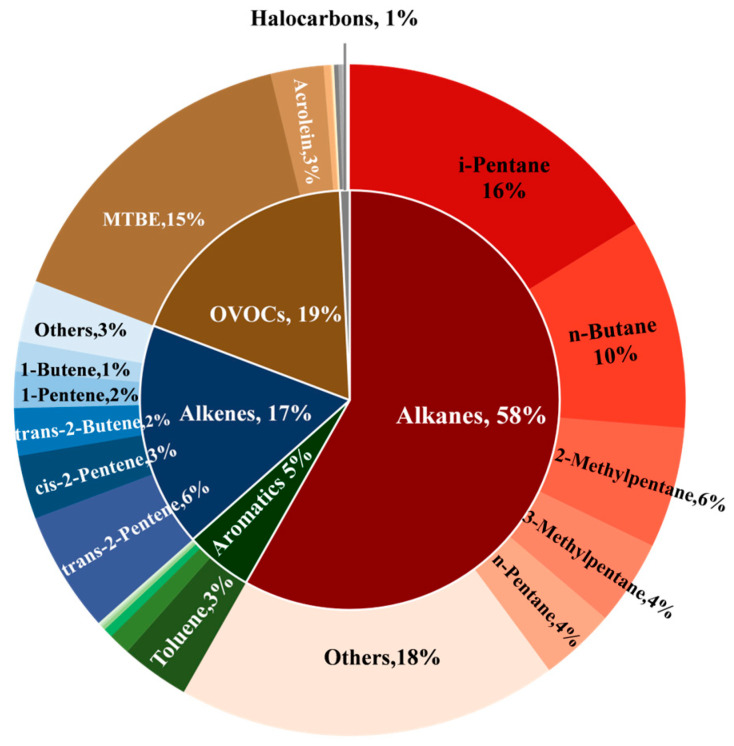
Contributions of VOC species to total VOC emissions from gas stations in Nanjing in 2023.

In 2023, total VOC emissions from gasoline and diesel evaporation at gas stations in Nanjing comprised 387 tons, including 344 tons from gasoline and 43.2 tons from diesel. As shown in Figure 4, alkanes were the dominant contributor to total VOC emissions (59%), followed by OVOCs (19%), alkenes (17%), aromatics (5%), and halocarbons (1%). The major species included i-pentane (16%), MTBE (15%), n-butane (10%), trans-2-pentene (6%), 2-methylpentane (6%), n-pentane (4%), cis-2-pentene (3%), and toluene (3%).

**Figure 5:**
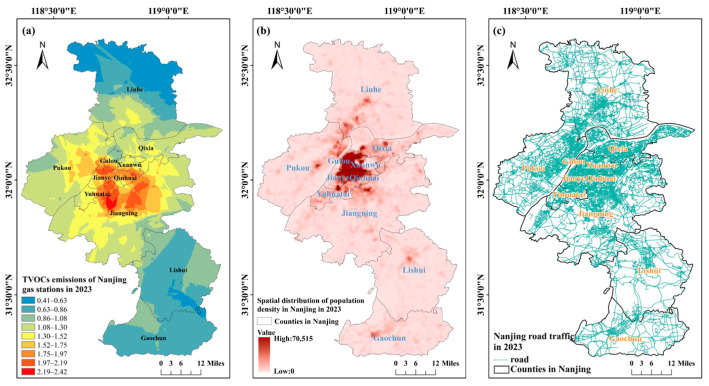
Spatial distributions of (**a**) VOC emissions from gasoline and diesel evaporation at gas stations, (**b**) population density, and (**c**) the road network in Nanjing in 2023.

Figure 5 shows the spatial distribution of total VOC emissions from gas stations, population density (obtained from the LandScan Global Population Database developed by Oak Ridge National Laboratory), and road network (sourced from OpenStreetMap) in Nanjing in 2023. High VOC emissions were mainly concentrated in the central urban districts, including Gulou, Xuanwu, Qinhuai, and Jianye, and decreased toward suburban areas. This spatial pattern is consistent with the distributions of population density and the road network, with VOC emissions showing a significant positive correlation with population density at the 1 km grid scale (*p* < 0.01). The co-location of high VOC emissions and densely populated areas may increase potential health risks, while proximity to major road networks may promote interactions with traffic-related emissions, therefore enhancing the formation of O_3_ and PM_2.5_.

## 4. Discussion

### 4.1. Formation Potential of O_3_ and SOA from Gas Station VOC Emissions

**Figure 6:**
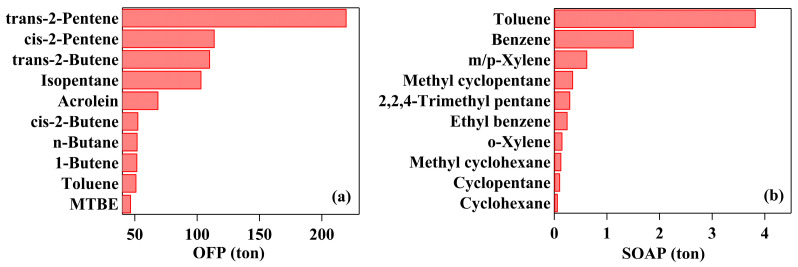
(**a**) Ozone formation potential (OFP) and (**b**) secondary organic aerosol formation potential (SOAP) of VOCs from gas stations in Nanjing in 2023.

The OFP and SOAP of VOC emissions from gas stations comprised 1203 tons and 7.73 tons, respectively. Figure 6a shows the top ten VOC species contributing to the total OFP from gas station emissions. The major contributors were C4–C5 alkenes (e.g., trans-2-pentene, cis-2-pentene, trans-2-butene), C4–C5 alkanes (e.g., i-pentane, n-butane), and OVOCs (e.g., acrolein, MTBE). This is highly consistent with the key contributing species identified in a recent study [56]. However, the OFP results from gas station emissions also differed from those for urban atmospheric VOCs in Nanjing [62], which identified toluene, ethylene, and m/p-xylene as major contributors. This discrepancy likely reflects additional contributions from petrochemical activities (e.g., ethylene, propylene) and solvent/coating use (e.g., toluene, xylenes).

Figure 6b presents the top ten VOC species contributing to SOAP from gas station emissions. The major contributors were aromatics (e.g., toluene, benzene, and m/p-xylene) and C5–C8 alkanes (e.g., methylcyclopentane, 2,2,4-trimethylpentane, and methylcyclohexane). These results are consistent with those reported for gas stations in Beijing [63], where aromatics and higher-carbon-number alkanes were also key contributors to SOAP.

### 4.2. Health Risk Assessment of VOC Emissions from Gas Stations

#### 4.2.1. Health Risk Assessment for Gas Station Workers

**Figure 7:**
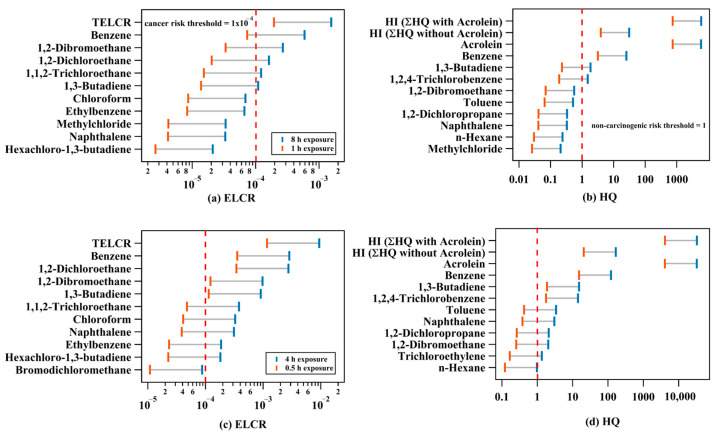
Excess lifetime cancer risk (ELCR) and hazard quotient (HQ) of VOCs for gas station workers: (**a**,**b**) gasoline; (**c**,**d**) diesel.

Figure 7 presents the calculated ELCR and HQ values for gas station workers exposed to VOCs from gasoline and diesel evaporation. The TELCR values ranged from 1.95 × 10^−4^ to 1.56 × 10^−3^ for VOCs from gasoline and 1.17 × 10^−3^ to 9.40 × 10^−3^ for VOCs from diesel, exceeding the acceptable cancer risk threshold of 1 × 10^−4^ [64], indicating potential carcinogenic risks for gas station workers. Among VOCs from gasoline, benzene exhibited the highest ELCR, followed by 1,2-dibromoethane, 1,2-dichloroethane, 1,1,2-trichloroethane, and 1,3-butadiene, all of which exhibited ELCR values exceeding the 1 × 10^−4^ threshold. For VOCs from diesel, ELCRs for nine species exceeded the 1 × 10^−4^ threshold, with benzene remaining the dominant contributor, followed by 1,2-dichloroethane, 1,2-dibromoethane, 1,3-butadiene, 1,1,2-trichloroethane, chloroform, naphthalene, ethylbenzene, and hexachloro-1,3-butadiene.

For non-carcinogenic risk, the HI values ranged from 7.45 × 10^2^ to 5.96 × 10^3^ for gasoline VOCs and from 4.08 × 10^3^ to 3.26 × 10^4^ for diesel VOCs. Given the much higher HQ of acrolein, HI was recalculated after excluding acrolein to better show the contributions of other VOCs. Even after this exclusion, the HI values remained at 3.90–31.2 for gasoline and 20.7–166 for diesel, still far exceeding the acceptable threshold of 1, indicating substantial non-carcinogenic risks. Acrolein exhibited the highest HQ in both gasoline and diesel VOCs (7.41 × 10^2^–5.93 × 10^3^ and 4.06 × 10^3^–3.25 × 10^4^, respectively), followed by benzene, 1,3-butadiene, 1,2,4-trichlorobenzene, 1,2-dibromoethane, toluene, 1,2-dichloropropane, and naphthalene.

Similar to the present study, previous studies in Zhengzhou [56] and Guangzhou [55] reported that VOC exposure from fuel vapors resulted in TELCR values of 4.6 × 10^−4^ and 1.3 × 10^−4^, and HI values of 5.9 and 3.1 for gas station workers, respectively, both exceeding the corresponding acceptable risk thresholds, indicating potential carcinogenic and non-carcinogenic risks to workers. Previous studies identified 1,3-butadiene and 1,2-dichloropropane as the main contributors to health risks [55,56]. Similarly, this study also found elevated risks associated with these species. However, unlike previous studies, benzene contributed most to carcinogenic risk, while acrolein contributed most to non-carcinogenic risk.

#### 4.2.2. Health Risk Assessment for Residents

**Figure 8:**
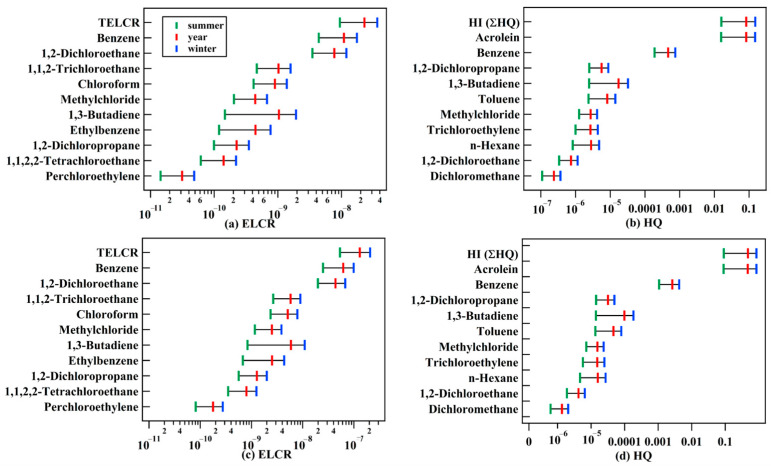
Excess lifetime cancer risk (ELCR) and hazard quotient (HQ) of VOCs from gas stations for residents: (**a**,**b**) entire city; (**c**,**d**) downtown area.

Figure 8 presents the calculated ELCR and HQ values for Nanjing residents exposed to VOC emissions from gas stations under annual average, summer, and winter conditions, for both the entire city and the central urban area. As shown in Figure 8a, the annual average, summer, and winter TELCR for Nanjing residents ranged from 9.43 × 10^−9^ to 3.64 × 10^−8^, all below the U.S. EPA-recommended minimum cancer risk threshold of 1 × 10^−6^ [64]. The corresponding HI values ranged from 0.016 to 0.150, also below the threshold of 1 [45]. These results suggest that, assuming VOCs were uniformly distributed across Nanjing, gas station emissions resulted in negligible health risks to residents. Similar findings were reported for Zhengzhou, where HI values were also below 1 and TELCR values were in the order of 10^−6^ [65].

For residents in the central urban area, TELCR increased to 5.36 × 10^−8^–2.07 × 10^−7^, while HI increased to 0.09–0.854. Although these values remain below health risk thresholds, the co-location of gas stations and densely populated areas suggests that health risks to nearby residents should not be overlooked. Additionally, the box model used in this study assumes instantaneous and uniform mixing of VOCs over the large study area and neglects spatial heterogeneity in gas station emissions. As a result, VOC concentrations and the associated health risks for residents near gas stations may be underestimated. Under unfavorable meteorological conditions, such as low wind speed, stable atmospheric stratification, and a reduced mixing-layer height, local accumulation of VOCs may further increase health risks for nearby residents.

### 4.3. Study Limitations and Uncertainties

In this study, VOC sampling at gas stations was conducted from July to September and included only three large gas stations. Evaporative VOC profiles may vary with temperature, fuel formulation, refueling activity, and station operating conditions. Therefore, the VOC chemical profiles of gasoline evaporation obtained in this study should be regarded as representative of summer evaporation conditions rather than year-round averages. This limitation may introduce uncertainty when extrapolating the source profiles and emission estimates to other seasons. Future studies with more sampling gas stations, broader seasonal coverage, and larger sample sizes are needed to further improve the representativeness and robustness of the results. In addition, because the gasoline and diesel evaporation samples were collected during actual refueling, the observed VOCs at gas stations may be influenced by ambient air mixing, nearby combustion emissions, or other background sources. Considering that halocarbons and acrolein are not typical constituents of gasoline or diesel evaporation, their trace detection in this study may be associated with minor fuel impurities, ambient air mixing, or interference from nearby sources.

## 5. Conclusions

This study established a 1 km × 1 km grid-based VOC emission inventory for gas stations by calculating VOC emissions from individual stations and applying Kriging spatial interpolation. The results show that VOC emissions from gasoline and diesel evaporation in Nanjing in 2023 were 344 tons and 43.2 tons, respectively. Emission profiles were characterized based on measured VOC compositions. Gasoline VOC emissions were dominated by alkanes (77%), with i-pentane and n-butane as the major contributors, together comprising 48%. In contrast, diesel VOCs exhibited a lower alkane fraction (44%) and higher fractions of OVOCs and aromatics, with MTBE as the largest contributor (24%). VOC emissions from gas stations were mainly concentrated in traffic-intensive central urban areas and exhibited a significant positive correlation with population density. Alkenes (particularly trans-2-pentene, trans-2-butene, and cis-2-pentene) contributed most to OFP, whereas aromatics (especially toluene, benzene, and m/p-xylene) dominated SOAP. Health risk assessment indicates that gasoline and diesel VOC emissions showed potential carcinogenic and non-carcinogenic risks to gas station workers. Benzene exhibited the highest carcinogenic risk, and acrolein contributed most to the non-carcinogenic risk. For residents, the overall carcinogenic and non-carcinogenic risks associated with gas station VOC emissions remained within acceptable levels, although elevated risks may still occur in densely populated areas or under unfavorable meteorological conditions.

## Figures and Tables

**Table 1:** VOC emission control efficiency (*η*, %) and emission factors (*EF*, mg·L^−1^) for different emission sources at gas stations.

Emission Sources	*UEF*	Type I S1 + S2		Type II S1 + S2 + OMS		Type III S1 + S2 + OMS + VRD	
		*η*	*EF*	*η*	*EF*	*η*	*EF*
(1) Tanker unloading	924	94.1	54.5	97.0	27.7	97.2	25.9
(2) Vehicle refueling *	768	76.5	179	88.1	90.9	93.0	53.5
(3) Tank breathing	91.0	91.2	8.01	95.5	4.10	97.2	2.55
(4) Nozzle dripping	73.0	14.7	62.3	22.4	56.6	30.1	51.0
(5) Hose permeation	7.00	0	7.00	0	7.00	0	7.00
Total	1959	82.9	311	89.9	186	92.5	140

* Vehicle refueling *UEF* = *UEF* without ORVR systems (1008) × 0.75 + *UEF* with ORVR systems (50) × 0.25 = 768 mg·L^−1^. Notes: OMS, Online Monitoring System; VRD, Vapor Recovery Device; ORVR, Onboard Refueling Vapor Recovery.

## Data Availability

Researchers wishing to access the data used in this study can make a request to the corresponding author: wangming@nuist.edu.cn.

## Supplementary Materials

The following supporting information can be downloaded at: https://www.mdpi.com/article/10.3390/toxics14060486/s1, Table S1: The UR and RfC values used for health risk assessment of individual VOC species; Table S2: The average mixing ratios (ppbv) of VOC species with relatively high health risks from gasoline and diesel evaporation.
