# Supplementary material for: A High-Resolution VOC Emission Inventory for Gas Stations in a Typical Yangtze River Delta City: Implications for Ozone Formation, Secondary Organic Aerosol Formation, and Health Risks
Source: Toxics. 2026 Jun 1;14(6):486. doi: 10.3390/toxics14060486 (PMC13307540; doi:10.3390/toxics14060486)
Supplement: Supplementary file 1 [file toxics-14-00486-s001.zip › toxics-4302111-supplementary.pdf]

Table S1 The UR and RfC values used for health risk assessment of individual VOC species

Species	UR ($mg^{-1}\cdot m^3$)	Source	RfC ($mg\cdot m^{-3}$)	Source
Ethane	0		0	
Propane	0		0	
Isobutane	0		0	
n-Butane	0		0	
Cyclopentane	0		0	
Isopentane	0		0	
n-Pentane	0		0	
2,2-Dimethyl butane	0		0	
2,3-Dimethyl butane	0		0	
2-Methyl pentane	0		0	
3-Methyl pentane	0		0	
Cyclohexane	0		0	
Methyl cyclopentane	0		0	
n-Hexane	0		0.7	IRIS
2,3-Dimethyl pentane	0		0	
2,4-Dimethyl pentane	0		0	
2-Methyl hexane	0		0	
3-Methyl hexane	0		0	
Methyl cyclohexane	0		3	HEAST 97
n-Heptane	0		0	
2,2,4-Trimethyl pentane	0		0	
2,3,4-Trimethyl pentane	0		0	
2-Methyl heptane	0		0	
3-Methyl heptane	0		0	
n-Octane	0		0	
n-Nonane	0		0	
n-Decane	0		0	
n-Undecane	0		0	
Benzene	0.0078	IRIS	0.003	Cal 14
Toluene	0		0.42	Cal 20
Styrene	0.00057	HEAST 91	1	IRIS
m/p-Xylene	0		0	
o-Xylene	0		0	
Ethyl benzene	0.0025	Cal 11	0	
1,2,3-Trimethyl benzene	0		0.06	IRIS
1,2,4-Trimethyl benzene	0		0.06	IRIS
1,3,5-Trimethyl benzene	0		0.06	IRIS
o-Ethyl toluene	0		0	
1,3-Ethyl toluene	0		0	
1,4-Ethyl toluene	0		0	
Isopropyl benzene	0		0.4	IRIS
n-Propyl benzene	0		0	

1,3-Diethyl benzene	0		0	
1,4-Diethyl benzene	0		0	
Naphthalene	0.034	Cal 11	0.003	IRIS
Dichloromethane	0.000013	IRIS	0.6	IRIS
Bromodichloromethane	0.037	Cal 15b	0	
Chlorodibromomethane	0		0	
Freon11	0		0.7	HEAST 97
Freon12	0		0.1	USEPA 14
Chloroform	0.023	IRIS	0.3	Cal 14
Methyl chloride	0.0018	HEAST 97	0.09	IRIS
Carbontetrachloroide	0.006	IRIS	0.04	Cal 14
Bromoform	0.0011	IRIS	0	
Methyl bromide	0		0.005	IRIS
1,1,1-Trichloroethane	0		1	Cal 14
1,1,2,2-Tetrachloroethane	0.058	Cal 11	0	
1,1,2-Trichloroethane	0.016	IRIS	0	
1,1-Dichloroethane	0.0016	Cal 11	0.5	HEAST 97
1,1-Dichloroethylene	0		0.2	IRIS
1,2-Dichloroethane	0.026	IRIS	0.4	Cal 14
1,2-Dibromoethane	0.6	IRIS	0.0008	Cal 14
trans-1,2-Dichloroethene	0		0	
Freon114	0		0	
Ethyl chloride	0		0	
Vinyl chloride	0.0088	IRIS	0.1	IRIS
Trichloroethylene	0.0048	IRIS	0.002	ATSDR
cis-1,2-Dichloroethylene	0		0	
perchloroethylene	0.0061	Cal 18	0.04	ATSDR
1,2-Dichloropropane	0.01	Cal 15b	0.004	IRIS
trans-1,3-Dichloropropene	0		0	
cis-1,3-Dichloropropene	0		0	
Hexachloro-1,3-butadiene	0.022	IRIS	0	
1,2,4-Trichlorobenzene	0		0.002	USEPA 14
1,2-Dichlorobenzene	0		0.2	HEAST 97
1,3-Dichlorobenzene	0		0	
1,3-Dichlorobenzene	0.011	Cal 11	0.8	IRIS
Chlorobenzene	0		1	Cal 14
Benzylchloride	0.049	Cal 11	0	
Ethene	0		0	
Acetylene	0		0	
Propene	0		3	Cal 14

1,3-Butadiene	0.03	IRIS	0.002	IRIS
1-Butene	0		0	
trans-2-Butene	0		0	
cis-2-Butene	0		0	
1-Pentene	0		0	
trans-2-Pentene	0		0	
cis-2-Pentene	0		0	
Isoprene	0		0	
1-Hexene	0		0	
Carbon disulfide	0		0.7	IRIS
Acetone	0		31	ATSDR
Acrolein	0		0.00002	IRIS
Isopropyl alcohol	0		0	
2-Butanone	0		5	IRIS
Tetrahydrofuran	0		2	IRIS
Ethyl acetate	0		0	
Vinyl acetate	0		0.2	IRIS
Methyl methacrylate	0		0.7	IRIS
MTBE	0		0	

Table S2 The average mixing ratios (ppbv) of VOC species with relatively high health risks from gasoline and diesel evaporation

Species	Gasoline (pbv)	Diesel (ppbv)
1,1,2,2-Tetrachloroethane	0.29	3.43
1,1,2-Trichloroethane	12.3	76.8
1,2,4-Trichlorobenzene	3.55	67.1
1,2-Dibromoethane	0.51	3.72
1,2-Dichloroethane	13.5	457
1,2-Dichloropropane	2.51	31.9
1,3-Butadiene	14.6	240
Acrolein	453	4967
Benzene	207	2004
Bromodichloromethane	0.65	6.18
Chloroform	5.40	51.2
Dichloromethane	12.1	205
Ethylbenzene	53.9	302
Hexachloro-1,3-butadiene	0.79	13.5
Naphthalene	1.64	30.5
n-Hexane	415	3389
Perchloroethylene	0.46	7.37
Toluene	504	6611
Trichloroethylene	0.56	8.74
